# Controlling Inflammation Pre-Emptively or at the Time of Cutaneous Injury Optimises Outcome of Skin Scarring

**DOI:** 10.3389/fimmu.2022.883239

**Published:** 2022-05-27

**Authors:** Sara Ud-Din, Ardeshir Bayat

**Affiliations:** ^1^ Plastic and Reconstructive Surgery Research, National Institute for Health Research (NIHR) Manchester Biomedical Research Centre, University of Manchester, Manchester, United Kingdom; ^2^ Medical Research Council - South Africa (MRC-SA) Wound Healing Unit, Division of Dermatology, University of Cape Town, Cape Town, South Africa

**Keywords:** skin scarring, wound healing, hypertrophic scars, keloid scars, inflammation, skin priming, pre-emptive skin priming

## Abstract

Inflammation plays an active role during the wound healing process. There is a direct association between the extent of injury as well as inflammation and the amount of subsequent cutaneous scarring. Evidence to date demonstrates that high levels of inflammation are associated with excessive dermal scarring and formation of abnormal pathological scars such as keloids and hypertrophic scars. In view of the multiple important cell types being involved in the inflammatory process and their influence on the extent of scar formation, many scar therapies should aim to target these cells in order to control inflammation and by association help improve scar outcome. However, most current treatment strategies for the management of a newly formed skin scar often adopt a watch-and-wait approach prior to commencing targeted anti-inflammatory therapy. Moreover, most of these therapies have been evaluated in the remodelling phase of wound healing and the evaluation of anti-inflammatory treatments at earlier stages of healing have not been fully explored and remain limited. Taken together, in order to minimise the risk of developing a poor scar outcome, it is clear that adopting an early intervention prior to skin injury would be optimal, however, the concept of pre-emptively priming the skin prior to injury has not yet been thoroughly evaluated. Therefore, the aim of this review was to evaluate the available literature regarding scar therapies that aim to target inflammation which are commenced prior to when a scar is formed or immediately after injury, with a particular focus on the role of pre-emptive priming of skin prior to injury in order to control inflammation for the prevention of poor scarring outcome.

## Introduction

The wound healing process is highly complex and is comprised of a series of well-defined stages that include inflammation, proliferation, and remodelling/scar formation (1). After an initial injury, clotting occurs and invading microbes activate an inflammatory response that is spread by the local release of chemotactic factors (2). There are multiple key players in the inflammatory process including neutrophils, monocytes, and other immune cells that are recruited to the wound site to clear cell debris and infections and aid in the tissue repair response (1–3). The length of response and the degree vary which has an effect on the final outcome. Certainly, there are significant benefits of producing an inflammatory response, but there are also negative aspects which can lead to non-healing wounds and excessive scarring, including hypertrophic scar and keloid formation (4–6). Many studies have suggested a direct association between the extent of injury/inflammation and the amount of scarring and there has been evidence that high levels of inflammation are associated with excessive scarring or development of abnormal scars, whereas inflammation is significantly diminished in wounds that heal without scars (7–11).

**Figure 1:**
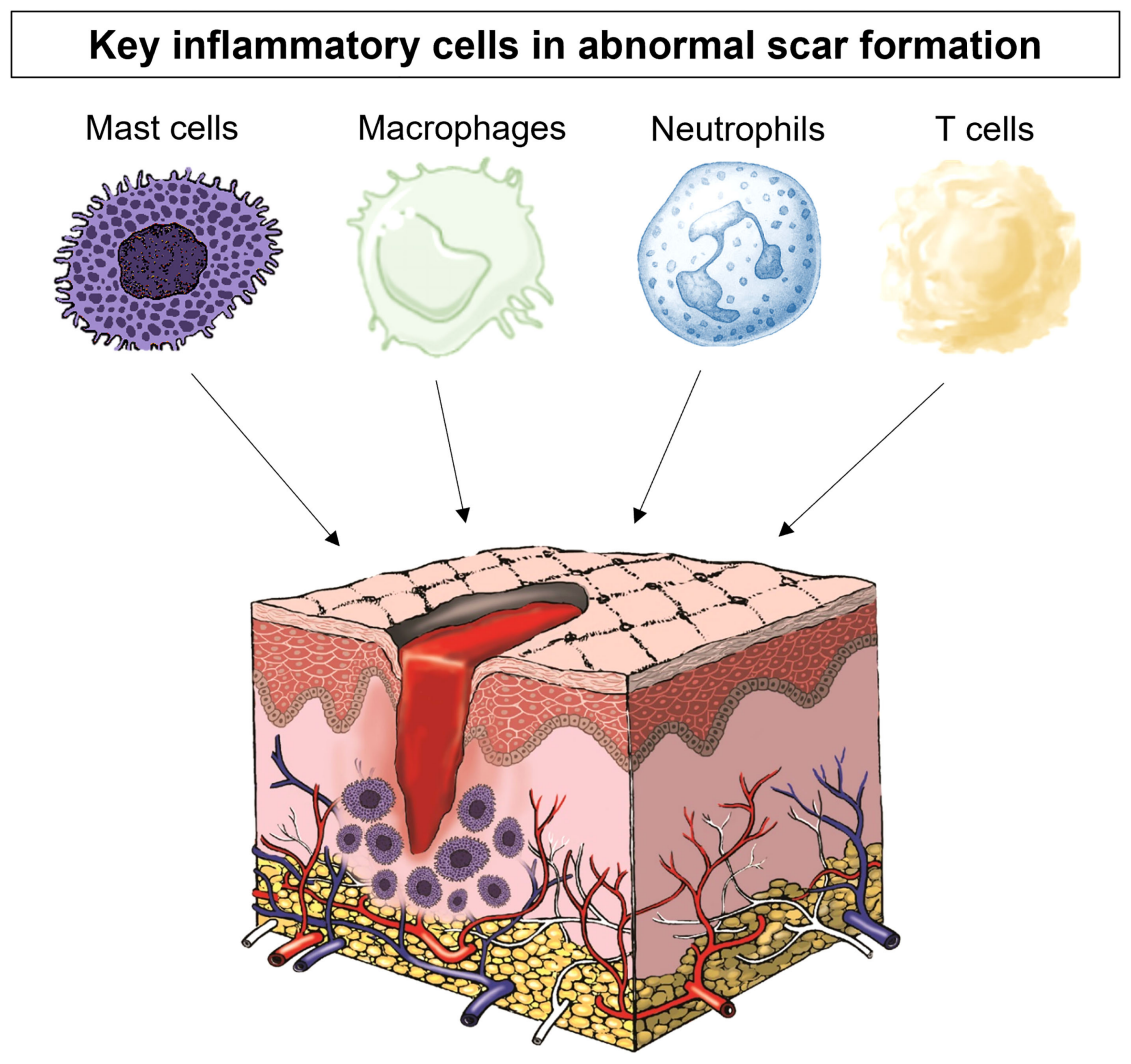
The key inflammatory cells in scar formation include mast cells, macrophages, neutrophils and T cells.

A number of immune cells have been associated with scar formation, including mast cells, macrophages and neutrophils as well as many inflammatory mediators have been shown to influence scar formation (12, 13) (**Figure 1**). In particular, macrophages, T cells, and mast cells have all been shown to be increased in abnormal scar tissue in particular in keloid, although in varying degrees. In keloid tissue, macrophages have been shown to have upregulated M2-associated genes which are highly relevant to tissue repair and remodelling (12, 13). While macrophages are indeed important for tissue repair, an overabundance on the other hand can be detrimental (14, 15). Current research has shown that T cells have a complex role in regulating scar formation, mainly due to the diverse T cell subsets (16, 17). Coculture of keloid fibroblasts with a Treg-enriched condition demonstrated reduced collagen synthesis in comparison to keloid fibroblasts cocultured with a Treg deficient T cell population (18). Neutrophils are the earliest leukocytes to arrive on the site of injury and they have been identified as the key cells in preventing microbes spreading and have a role in fibrosis (19) but there is a lack of *in vivo* evidence of the role of neutrophils in scar formation. Mast cells, however, are important in hypersensitivity responses and allergic reactions and their link with scar formation has also been studied (8, 20–22). Observations of their numbers and activation status were positively correlated with the degree of scar formation (23, 24). They have been identified as being significantly increased in hypertrophic and keloid scars (8). Furthermore, our group has shown that not only is this a problem in excessive scarring but there are high numbers of mast cells in normal scarring compared to normal skin and this number is further increased after injury with levels not returning to normal even by week 8 post-injury (22).

**Figure 2:**
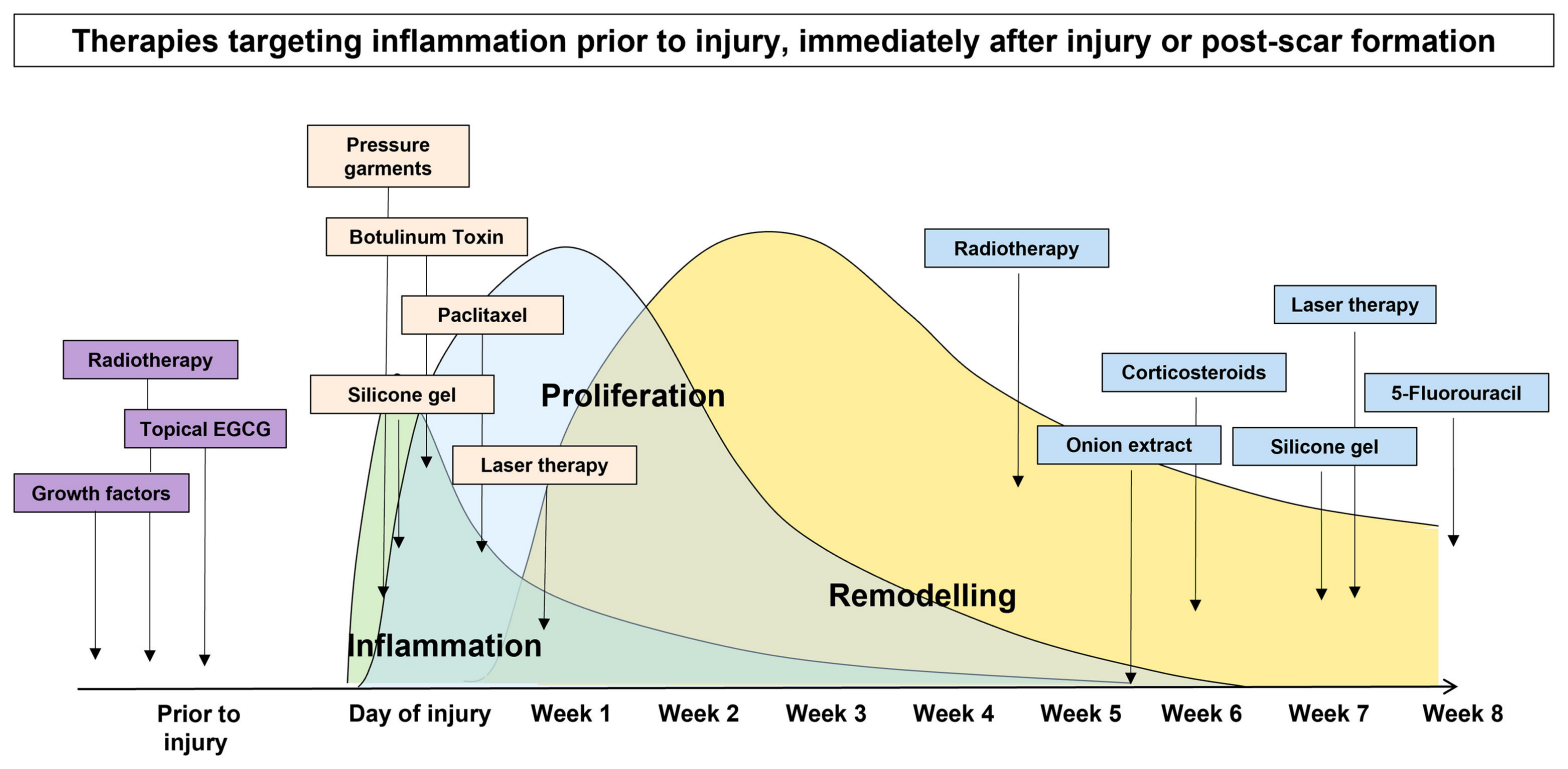
Scar therapies which aim to target inflammation pre-emptively prior to injury, immediately after injury and post-scar formation.

In view of these important cell types being involved in the inflammatory process and their influence on the extent of scar formation, many scar therapies aim to target these cells in order to quench inflammation to improve scar outcome (25–29). However, most of these therapies have been evaluated in the remodelling phase of wound healing mainly due to the scar being most evident at this stage and the amount of inflammation is most noticeable when correlated to scar severity. Conversely, the evaluation of anti-inflammatory treatments at earlier stages (30, 31) have been limited, because there has been the assumption that inflammation in the early phase of healing is necessary and beneficial for infection prevention and neovasculature development in wound healing. Most current treatment strategies for the management of a newly formed skin scar often adopt a watch-and-wait approach prior to commencing targeted therapy (32, 33). However, in order to minimise the risk of developing a poor scar outcome, taking an early intervention prior to skin injury is desirable. Priming is defined as a substance that prepares the skin for use. Priming the skin prior to an invasive intervention for achieving an optimal result has been studied (34–40). However, the concept of pre-emptively priming the skin prior to injury has not been thoroughly evaluated. Therefore, the aim of this review was to evaluate the available literature regarding scar therapies which aim to target inflammation which are commenced after a scar has formed or immediately after injury, and we will particularly focus on the role of pre-emptive priming of skin prior to injury in order to control inflammation for the prevention of poor scarring outcome (**Figure 2**).

## Targeting Inflammation to Treat Existing Skin Scarring

A number of anti-inflammatory treatments for skin scarring have been evaluated for existing scars including radiotherapy, compression (pressure therapy), laser and 5-fluorouracil therapy (40–43). These therapies have been thought to supress inflammation by inhibiting angiogenesis as inflammatory cells migrate through blood (5). A non-pharmacological treatment that has been used is onion extract ointment which is composed of phenolic compounds. This active ingredient can be converted to quercetin which is an anti-inflammatory derivative with its effects including mast cell stabilisation and anti-proliferative effects (44). This treatment has been used post-operatively to compare the efficacy of silicone gel containing onion extract and aloe vera to silicone gel sheets to prevent postoperative hypertrophic scars and keloids (45). Another study used onion extract on C-Section scars 4 weeks post-surgery and noted improved scar pigmentation and pliability (46).

There are a number of pharmacological treatments which have been used to target inflammation for scarring. Steroids are well known for their anti-inflammatory effect and are widely used for treatment of autoimmune diseases. They decrease inflammation by suppressing the activities of both myeloid and lymphoid cells (47). As increased inflammation promotes excessive scarring, steroid administration is one of the most commonly used treatments for keloid and hypertrophic scars (47). An anti-cancer drug named, Paclitaxel has been shown to regulate inflammatory responses and fibrosis. It has been found to inhibit the NF-kB pathway (48) and attenuate fibrosis by blocking STAT-3 signaling (49). This anti-inflammatory and anti-fibrosis effect has also been observed in animal models of keloid (50) and hypertrophic scars (51). *In vitro* studies have shown that the expression of IL-6 and TNF-a, as well as the production of a-SMA and collagen I, decreased in human keloid fibroblasts following paclitaxel administration (50).

Laser therapies have been used to target inflammation for existing scars. One study used a diode laser with an intralesional optical fibre delivery device in the treatment of hypertrophic and keloid scars and showed a significant reduction in pigmentation and blood perfusion levels (52). A PDL/Nd : YAG laser has also been used in the treatment of surgical scars and the short-term effects were evaluated by *in vivo* confocal microscopy and the long-term effects by clinical assessment of the scars appearance (53). The results demonstrated scar improvements with the laser treatment by showing lower numbers of vessels and decreased amount of collagen fibers.

## Targeting Inflammation to Treat Scars Immediately Post-Injury

A number of studies have aimed to explore the effects of early intervention of inflammation on scar formation with the outcomes varying by different approaches. Surgery, tapes, sheets, and pressure garments are considered useful in decreasing inflammation in the wound area by reducing the degree of tension at the wound edge and all have been used immediately during or after surgery/injury (54). Silicone gel is considered the first line of treatment for most scar management cases, including hypertrophic and keloid scars (55). This anti-scar activity could be partly attributed to the occlusive environment created by silicone gel dressings, and hydration is also believed to lead to the stabilisation of mast cells (16). This treatment is usually applied early once a scar has formed.

Laser therapies have been used both on existing scars and earlier in the healing process. In most studies, the recommended time frame for commencing scar treatment using fractional CO2 laser has remained relatively consistent at more than 2 months after surgery (56). However, recent studies have emphasised the importance of early treatment to reduce scar formation using fractional CO2 laser (56, 57). Resurfacing of scars using fractional CO2 laser with early interventional treatment has been shown to reduce scar formation (58–61). A meta-analysis has supported the efficacy of lasers in minimizing closed surgical scars when treated less than 1 month after surgery (62). There has also been reports on the use of intraoperative fractional CO2 laser treatment of wound edges, which significantly improved the appearance and texture of the scars (63). Erbium: YAG laser treatment, performed immediately after surgery, can also improve the appearance of a surgical scar (64). Thus, it appears that early treatment to the wound may lessen scar formation. Non-ablative fractional laser treatment has also been directly used on the wounded skin barrier immediately after punch biopsy collection, in order to improve scar appearance (65).

Botox which is the commercial name of botulinum toxin (BTX), a neurotoxin produced by clostridium botulinum has been shown to have anti-inflammatory effects on wound healing and scar formation (4, 66, 67). A meta-analysis evaluating intralesional injection of BTX-A versus corticosteroids and placebo in the treatment of hypertrophic scars and keloids showed that BTX-A was most effective (68). Another study compared the effects of inflammation intervention in earlier phases (on the day of operation) with that in later phase (2-week postoperatively) on scar formation after thyroidectomy (31). It has been suggested that by injecting BTX-A and its diffusion into the surrounding muscles at different times during the wound healing process this may have different effects on scar cosmesis by reducing the levels of inflammatory cytokines and mechanical tension on the wound edges (69–71). Although no difference was observed in scar size, early application of BTX-A achieved better scar appearance in relation to erythema, skin elasticity and patient satisfaction, compared to later application. However, the authors did not perform histopathologic assessments in order to evaluate the changes in inflammatory cell infiltration during specific phases of inflammation and structural changes of the skin.

Another study evaluated the effectiveness of intraoperative electro-abrasion for scar revision (72). This was a prospective, randomized, observer-blinded, split-scar study with 24 linear scar segments from patients undergoing Mohs micrographic surgery. After placement of dermal sutures, half of the wound was randomly treated with electro-abrasion whilst the other half was used as the control. Results showed improved scar topography but worsened erythema.

## Pre-Emptive Priming of Skin Prior to Injury

Several studies support the concept of pre-emptive priming of skin prior to cutaneous injury (34–39). For instance, research on the treatment of pigmented acne scars by ablative laser therapy advocates the use of priming agents to reduce wound healing time, decrease the risk of post-inflammatory hyperpigmentation and provide ultraviolet damage protection (34). Additionally, radiotherapy has been used as an adjuvant therapy for the treatment of keloid scarring both prior to extralesional excision and post-surgery and this has been shown to lead to lower recurrence rates (73).

Another option to target inflammation is by way of topical therapies which have been thought to be a viable therapeutic strategy for restricting scar tissue production and enhancing the cosmetic and functional clinical outcomes resulting from skin injury (74). Our group has conducted two double-blind randomised controlled clinical trials in humans to evaluate the concept of immediate versus delayed application of a topical formulation post-surgical wounding in an excisional punch-biopsy model (75) and the concept of pre-emptive priming of the wound site compared to immediate or delayed application (76). The objective was to deliver an active compound at the optimal time post-surgically induced injury, in order to maximise its impact and improve healing. The results from the first trial (75) demonstrated reduced mast cell numbers, scar thickness and angiogenesis plus increased hydration and elasticity when an anti-scarring topical formulation was applied immediately to the zone of injury and with delayed application of topical two-weeks post-wounding. The topical formulation used epigallocatechin-3-gallate (EGCG) as the active ingredient. EGCG is the most potent anti-inflammatory anti-oxidant and active component and the most extensively studied green tea catechin (75). Our group have previously evidenced the transdermal delivery uptake of EGCG by using high-performance liquid chromatography and demonstrated this penetrated to the deep dermal tissue and remained in the dermis (74). The mechanism of action of EGCG in wound healing and scarring remain unclear but a number of *in vitro* studies have been conducted to identify the mechanism of action of EGCG on mast cells, angiogenesis and scar shrinkage (77–79).

**Figure 3:**
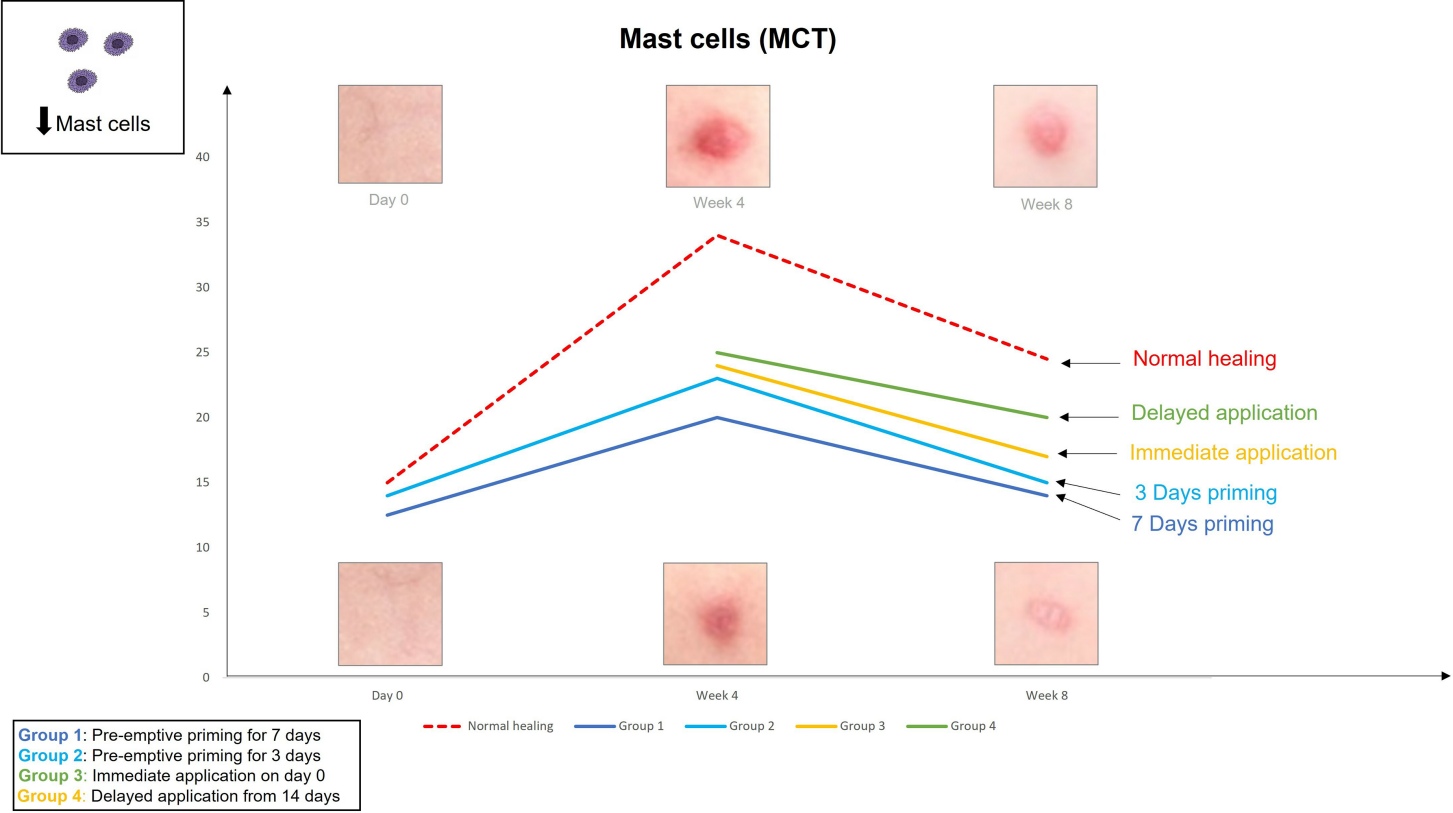
We evaluated the pre-emptive, immediate and delayed intervention of topical Epigallocatechin-3-gallate (EGCG) on skin scarring by conducting a randomsied double blind controlled trial on 40 healthy volunteers over 8 weeks. Here, we demonstrate graphical representations of the trends. Application of topical EGCG, particularly 7 days prior to injury demonstrated greater reductions in mast cells, compared to the other modes of topical application.

**Figure 4:**
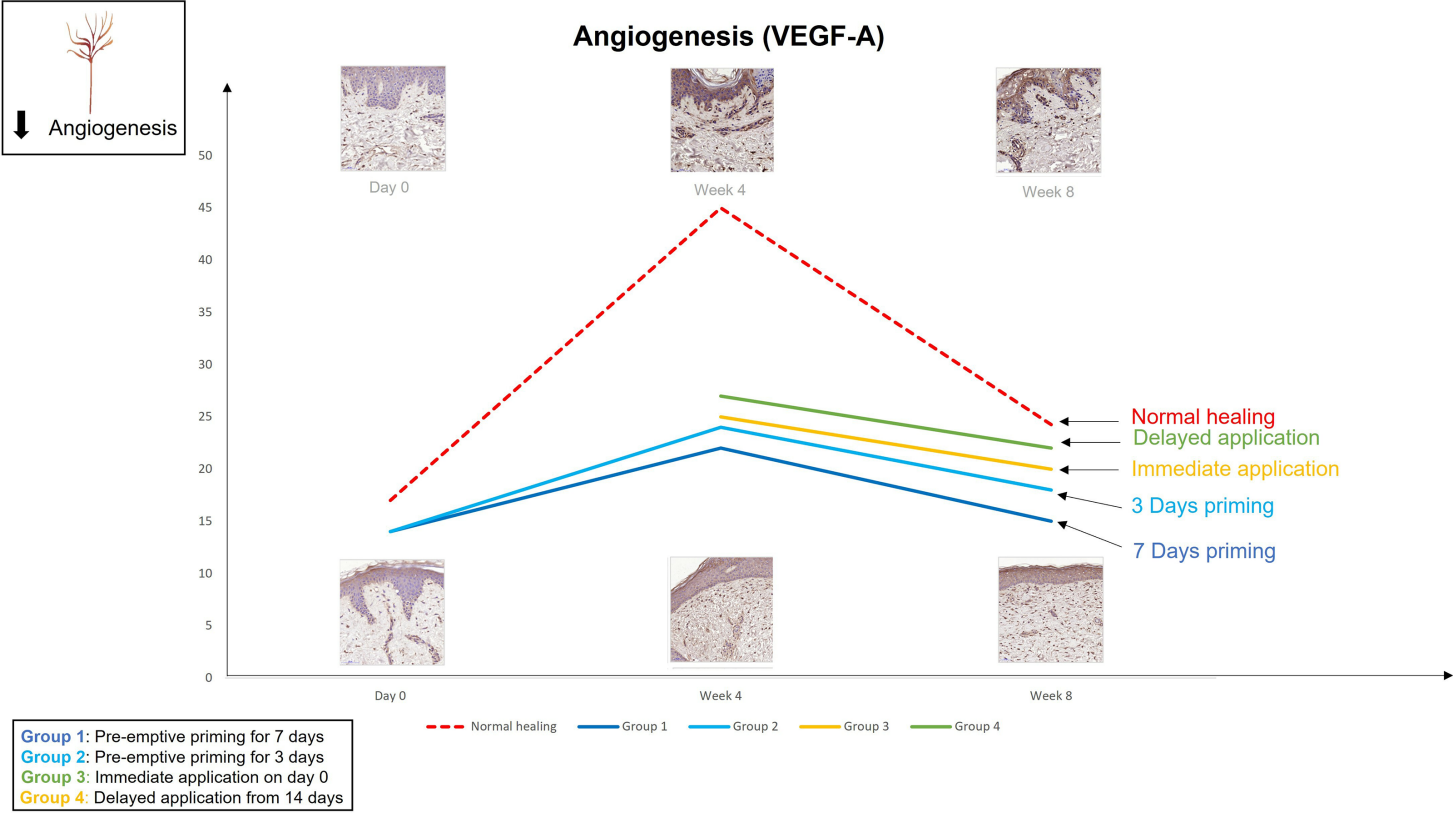
We evaluated the pre-emptive, immediate and delayed intervention of topical Epigallocatechin-3-gallate on skin scarring by conducting a randomsied double blind controlled trial on 40 healthy volunteers over 8 weeks. Graphical representation of the trends for angiogenesis (VEGFA) which demonstrates that pre-emptively priming the skin 7 days prior to injury shows greater reductions in VEGFA compared to all other modes of application.

**Figure 5:**
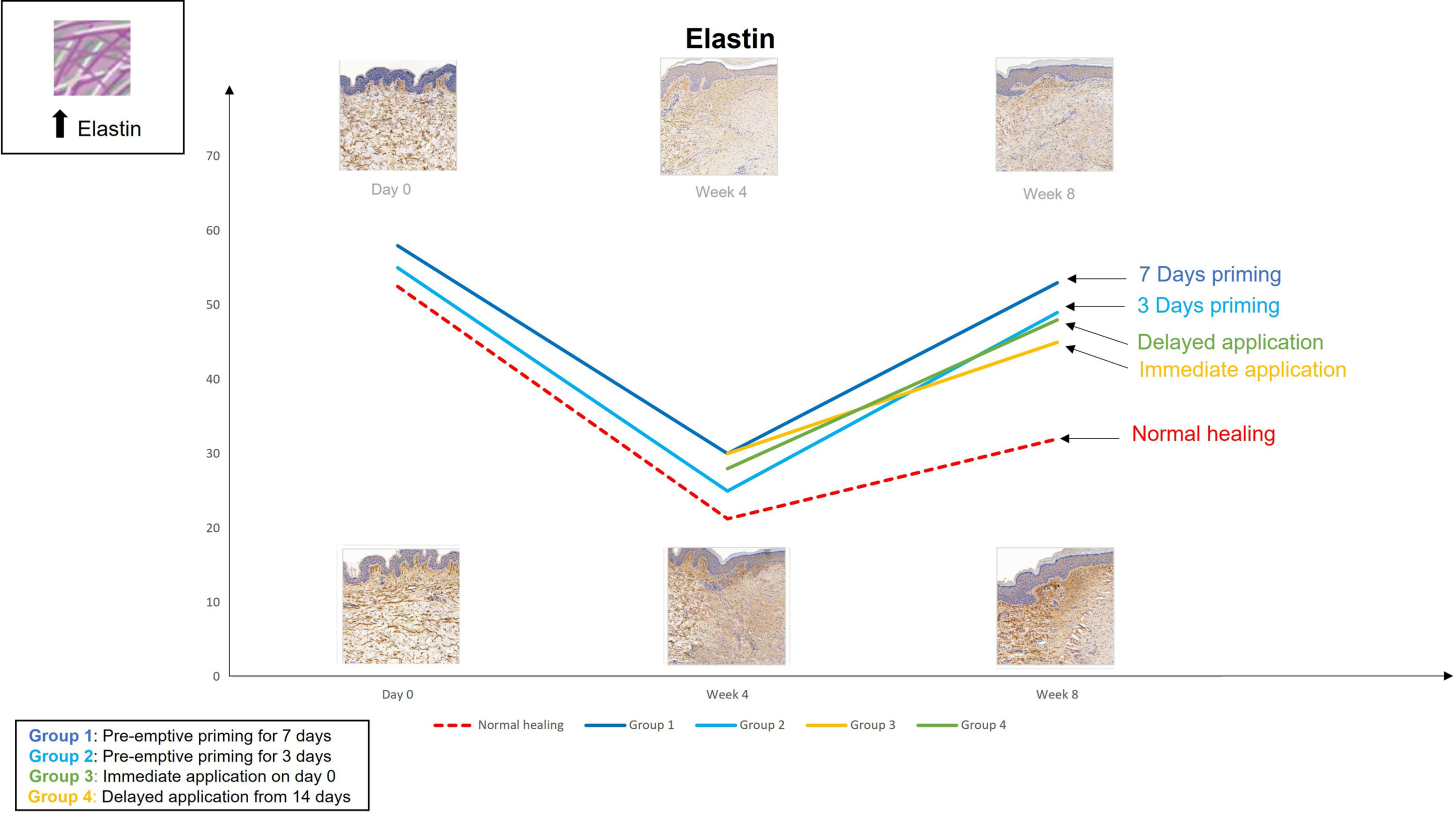
We evaluated the pre-emptive, immediate and delayed intervention of topical Epigallocatechin-3-gallate on skin scarring by conducting a randomsied double blind controlled trial on 40 healthy volunteers over 8 weeks. Graphical representation of the trends for elastin which demonstrates that pre-emptively priming the skin 7 days prior to injury shows greater increases in elastin compared to all other modes of application.

**Figure 6:**
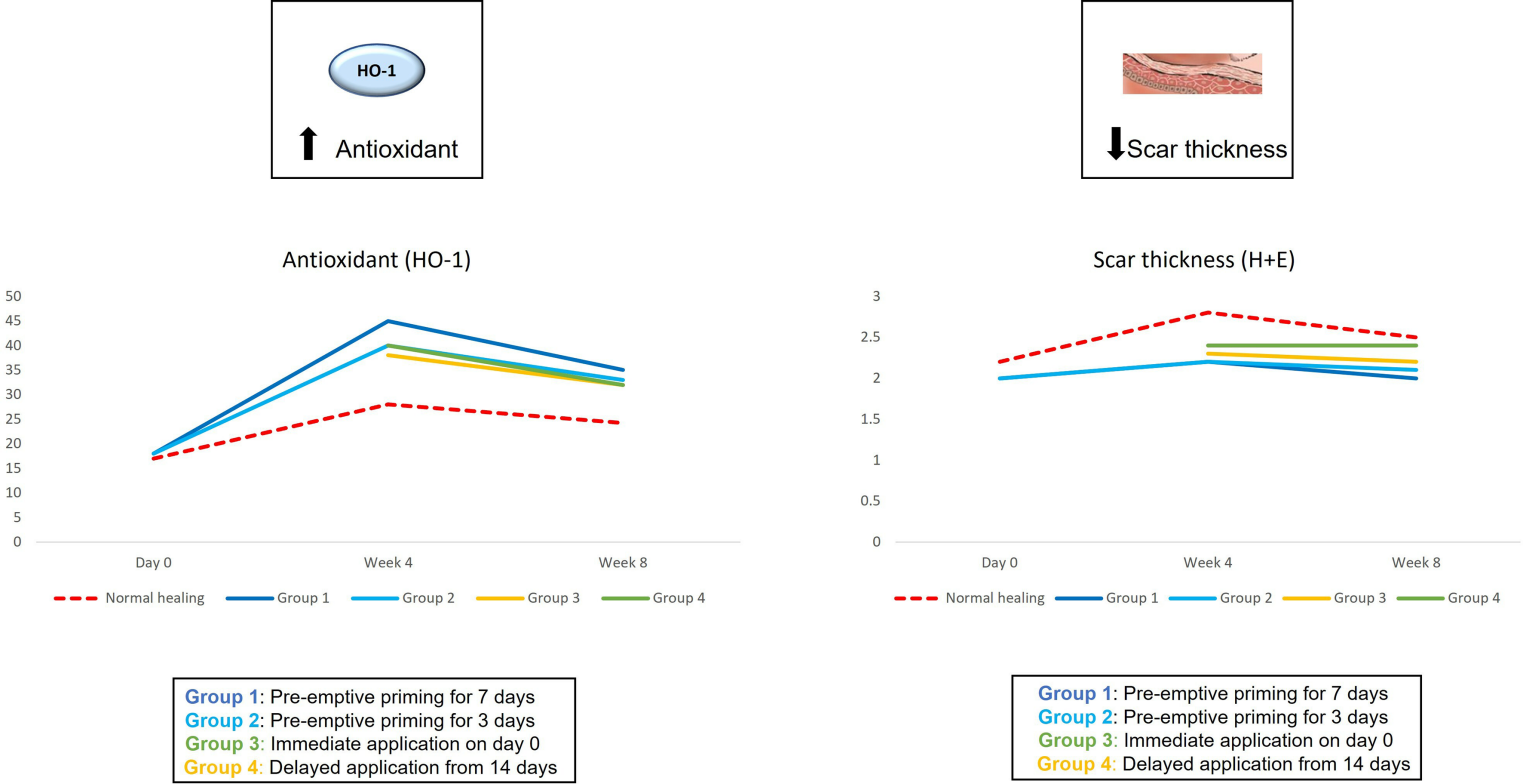
We evaluated the pre-emptive, immediate and delayed intervention of topical Epigallocatechin-3-gallate on skin scarring by conducting a randomsied double blind controlled trial on 40 healthy volunteers over 8 weeks. Graphical representation of the trends for antioxidant levels and skin thickness which demonstrated that pre-emptively priming the skin 7 days prior to injury shows greater reductions in skin thickness and greater increases in antioxidant levels compared to all other modes of application.

The findings from the first study provoked the hypothesis that even earlier application of an active such as EGCG could have beneficial effects on the outcome of scarring. Therefore, the second double-blind randomized placebo-controlled trial used various modes of the same anti-scarring topical formulation (containing EGCG) application utilising a full-thickness excisional surgical biopsy approach to identify whether pre-emptive priming pre-surgically induced injury had a greater impact on scarring outcome compared to immediate or delayed application (76). This demonstrated that the effects were further maximised by targeting the source of inflammation earlier. Based on our data, early intervention with the application of topical EGCG particularly 7 days prior to injury demonstrated greater reduction in mast cells (**Figure 3**) and angiogenesis (**Figure 4**), increases in elastin (**Figure 5**) and antioxidant levels and reductions in scar thickness (**Figure 6**) compared to the other modes of topical application. The reduction in mast cell numbers and subsequent lower levels of angiogenesis are beneficial as by targeting these cell types it is thought that this will contain inflammation to achieve a better scar outcome. Additionally, healing requires a strong angiogenic response but high angiogenesis also directly influences scar formation so partial inhibition of the angiogenic response could reduce scar formation or the potential for the development of abnormal skin scarring (78). Furthermore, animal studies have suggested that knocking out mast cells are not detrimental to acute wound healing and there have been a number of studies that have blocked mast cell activation to improve scar formation (80–82).

The potential therapeutic benefits of priming with proangiogenic factors and cells prior to surgery with a combination of proangiogenic growth factors for wound healing in normoglycemic and diabetic mice has been investigated (38, 83). Priming with a combination of VEGF, fibroblast growth factor (FGF) and PDGF has been shown to lead to more rapid closure times, higher vessel densities and better functional outcomes (38). In addition to proangiogenic growth factors, endothelial progenitor cells (EPCs) have presented a potential pre-treatment option for diabetic wounds (83). A murine study showed beneficial effects with pre-treatment by pro-angiogenic growth factors in the healing of diabetic incisional wounds (83). They demonstrated that priming with proangiogenic growth factors and EPCs enhanced incisional wound healing, as defined by a more rapid wound re-epithelialization, higher wound vascularization and higher tensile strength. In particular, the assessment of time-to-closure and functional outcome revealed an advantage for the groups primed with EPCs in comparison to the control animals. It is noted that while the 7 days pre-emptive treatment is feasible and beneficial for elective surgeries, this choice may not be available for traumatic injuries and emergency surgeries. Further research would be required to identify if application of this topical formulation for a short period of time, for example a number of hours prior to trauma surgery, could also be beneficial to the outcome of the scarring.

## Discussion

We cannot erase scarring, but we can modulate the course of healing, by reducing excessive inflammation, to achieve a better scar outcome. Normal wound healing requires inflammation and tissue remodelling mounting to appropriate degrees. Excessiveness of either of these two factors can lead to the development of keloid or hypertrophic scars. Particularly overabundance of inflammatory cells in the remodelling phase correlates with pathological scar formation, indicating the presence of a prolonged inflammatory phase overlapping with the later phases of healing. Inflammatory cell activation is vital for the prevention of infection in contaminated wounds particularly in the early stages of repair and can aid in cleaning the wound site and clearing debris (25, 26). Blocking the inflammatory pathway completely would be detrimental to this process therefore, instead of blocking, by reducing excessive inflammation this would allow infection prevention but not lead to abnormal scarring. However, blocking mast cell activation has been shown to not be detrimental to wound healing as there have been a number of studies that have blocked mast cells in order to improve scar formation (80–82). Furthermore, the concept of inducing altered inflammatory memory response in immune cells responding to injury or in tissue stem cells and epithelial cells has been studied. There have been suggestions that there are benefits in wound healing and tissue repair by priming these cell types to induce immune/inflammatory memory, characterized by epigenetic modifications, leading to increased responsiveness and more rapid inflammatory resolution upon secondary insult (84, 85). Further work could be carried out to elucidate the mechanisms involved in the therapeutic potential of inducing this inflammatory memory.

Early interventions targeting the source of inflammation have shown benefits in minimising scar formation. Many of the treatments used are general anti-inflammatory therapies, but more specific early interventions that target specific cell types such as reducing mast cells or supressing macrophage accumulation may be more optimal. Therefore, more research should be focussed on investigating the roles of the individual key players in inflammation and the effects of pre-emptive treatments to target these specific cell types to improve scar outcomes. By blocking certain pathways in the early phases of wound healing or prior to wounding this may have prophylactic effects for the prevention of abnormal skin scarring.

## Ethics Statement

The studies involving human participants were reviewed and approved by University of Manchester Research Ethics Committee. The patients/participants provided their written informed consent to participate in this study.

## Author Contributions

SU-D drafted the manuscript. AB developed the concept and edited the manuscript. All authors contributed to the article and approved the submitted version.

## Conflict of Interest

The authors declare that the research was conducted in the absence of any commercial or financial relationships that could be construed as a potential conflict of interest.

## Publisher’s Note

All claims expressed in this article are solely those of the authors and do not necessarily represent those of their affiliated organizations, or those of the publisher, the editors and the reviewers. Any product that may be evaluated in this article, or claim that may be made by its manufacturer, is not guaranteed or endorsed by the publisher.
